# Magnetic resonance imaging in central nervous system tuberculosis

**DOI:** 10.4103/0971-3026.57205

**Published:** 2009-11

**Authors:** Richa Trivedi, Sona Saksena, Rakesh K Gupta

**Affiliations:** Department of Radiodiagnosis, Sanjay Gandhi Post Graduate Institute of Medical Sciences, Lucknow - 226 014, UP, India

**Keywords:** Central nervous system, tuberculosis, magnetic resonance imaging

## Abstract

Tuberculosis (TB) in any form is a devastating disease, which in its most severe form involves the central nervous system (CNS), with a high mortality and morbidity. Early diagnosis of CNS TB is necessary for appropriate treatment to reduce this morbidity and mortality. Routine diagnostic techniques involve culture and immunological tests of the tissue and biofluids, which are time-consuming and may delay definitive management. Noninvasive imaging modalities such as computed tomography (CT) scan and magnetic resonance imaging (MRI) are routinely used in the diagnosis of neurotuberculosis, with MRI offering greater inherent sensitivity and specificity than CT scan. In addition to conventional MRI imaging, magnetization transfer imaging, diffusion imaging, and proton magnetic resonance spectroscopy techniques are also being evaluated for better tissue characterization in CNS TB. The current article reviews the role of various MRI techniques in the diagnosis and management of CNS TB.

## Introduction

Tuberculosis (TB), caused by *Mycobacterium tuberculosis*,[1] accounts for eight million annual, worldwide deaths. Involvement of the central nervous system (CNS) is one of the most serious forms of this infection and is responsible for a high mortality and morbidity. The pandemic of acquired immunodeficiency syndrome (AIDS) has resulted in an increased incidence of CNS TB worldwide.[2] Granulomatous inflammatory reaction in CNS caused by *M. tuberculosis* may involve the meninges, brain, spinal cord, and the bones covering the brain and spinal cord, and may manifest clinically depending on the specific location of the disease process.

## Cranial TB

### Meningitis

Tuberculous meningitis (TBM) is the most common cause of chronic meningitis, especially in developing countries. Infection may occur either by hematogenous seeding of the meninges or release of the organism into the meningeal space.

Diagnosis of TBM is made by cerebrospinal fluid (CSF) examination, which characteristically shows a lymphocytic pleocytosis, increased CSF protein, and decreased CSF sugar concentration.[3] CSF culture for acid-fast bacilli (AFB) and CSF polymerase chain reaction (PCR) are confirmatory tests for the diagnosis of TBM. The sensitivity of CSF culture for the detection of AFB has been reported to be approximately 50%.[4] CSF PCR examination is a newer technique, capable of amplifying minute amounts of DNA into millions of identical copies. It is more sensitive than the combination of microscopic examination and culture for *M. tuberculosis*.[5] Noninvasive imaging is essential as it helps in demonstrating the complications of TBM besides playing an important role in its diagnosis.

**Figure 1(a-h):**
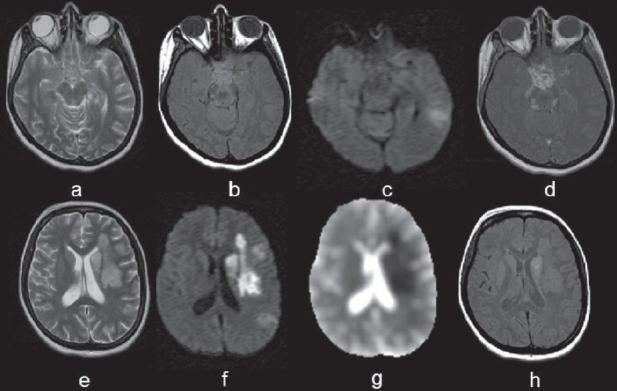
Tuberculous meningitis with vasculitis. T2W image (a) shows no apparent signal abnormality in the basal brain parenchyma. The corresponding magnetization transfer T1W image (b) shows hyperintensity in the perimesencephalic and supreseller cisterns. On DWI (c) restriction is noted in the left temporal region. The postcontrast T1W image (d) shows abnormal enhancement along the perimesencephalic and supraseller cisterns. In the same patient, axial sections at a higher level show hyperintensity on a T2W image (e) in the left middle cerebral artery (MCA) territory with hyperintensity on DWI (f) and low ADC values in the ADC map (g) suggesting a large left middle cerebral artery (MCA) territory infarct. The postcontrast T1W image (h) shows abnormal enhancement in the left caudate nucleus region

Common findings on imaging are abnormal meningeal enhancement in the basal cisterns, hydrocephalus, and vascular complications. Magnetic resonance imaging (MRI) scores over computed tomography (CT) scan in the early detection of meningeal pathologies.[6] During the early stages of the disease, noncontrast MRI studies usually show little or no evidence of any meningeal abnormality. With disease progression, swelling of the affected subarachnoid spaces occurs with associated mild shortening of T1 and T2 relaxation times in comparison with normal CSF. Postcontrast T1W images show abnormal meningeal enhancement, especially in the basal cisterns. Commonly involved sites are the interpeduncular fossa, pontine cistern, and the perimesencephalic and suprasellar cisterns [Figure 1]. Involvement of the sulci over the convexities and of the Sylvian fissures can also be seen.[7–9] Cerebellar meningeal and tentorial involvement is uncommon.

Magnetization transfer (MT) imaging is considered to be superior to conventional spin echo (SE) sequences for imaging abnormal meninges, which appear hyperintense on precontrast T1W MT images and show further enhancement on postcontrast T1W MT images.[10] In addition, MT ratio (MTR) quantification helps in predicting the etiology of the meningitis.[6,10,11] Visibility of the inflamed meninges on precontrast T1W MT images with low MTR is specific for TBM, helping in differentiating it from other nontuberculous chronic meningeal infections [Figure 1].[11] There is no published study of *in vivo* MRI spectroscopy (MRS) in TBM; however, *ex vivo* spectroscopy of CSF has been attempted in this context.[12] High-resolution *ex vivo* MRS of the CSF shows signals from Lac, acetate, and sugars along with cyclopropyl rings (−0.5 to +0.5 ppm) and phenolic glycolipids (7.1 and 7.4 ppm). These have not been observed in pyogenic meningitis. The combination of *ex vivo* MRS with MT MRI may be of value in the diagnosis of TBM. Complications that are secondary to TBM may either develop as the disease progresses or while the patient is on specific chemotherapy. The sequelae associated with TBM are discussed in detail below.

#### Hydrocephalus:

Hydrocephalus encountered in TBM can be broadly divided into two types: (1) communicating type, which is common, secondary to an obstruction of the basal cisterns by inflammatory exudates and (2) obstructive type, which is less common and either secondary to a focal parenchymal lesion causing mass effect or due to the entrapment of a part of the ventricle by granulomatous ependymitis.[13] Periventricular hyperintensity on proton density and T2W images is due to the seepage of the CSF fluid across the white matter and usually suggests hydrocephalus under pressure, which is an indication for CSF diversion surgery to decompress the ventricular system. Chronic hydrocephalus may result in atrophy of the brain parenchyma. Endoscopic third ventriculostomy is a procedure gaining acceptance as patency of the stoma can be demonstrated by CSF flow dynamics.[14]

#### Vasculitis:

It is a complication that is commonly seen at autopsy in cranial TBM.[13] The adventitial layer of small and medium-sized vessels develops changes similar to those of the adjacent tuberculous exudates. The intima of the vessels may eventually be affected or eroded by fibrinoid–hyaline degeneration. In later stages, the lumen of the vessel may get completely occluded by reactive subendothelial cellular proliferation.[15] Ischemic cerebral infarction resulting from the vascular occlusion is a common sequelae of tuberculous arteritis. The middle cerebral and lenticulostriate arteries are most commonly affected.[15,16] The conventional angiographic features of cranial TBM consist of a hydrocephalic pattern, narrowing of the arteries at the base of the brain, and narrowed or occluded small or medium-sized arteries.[17] MRI angiography (MRA) may help in the detection of vascular occlusion. High-field MRA with contrast is more sensitive than conventional MRA in the detection of occlusion of smaller vessels that are more commonly involved by the pathology. It has been reported that the incidence of infarcts detected by CT scan varies from 20.5 to 38%. However, MRI detects more infarcts, including hemorrhagic infarcts, than does CT scan.[18] The majority of the infarcts are in the basal ganglia and internal capsule due to the involvement of the lenticulostriate arteries.[16,19] Diffusion-weighted imaging helps in the early detection of this complication [Figure 1].[19]

## Focal or Diffuse Pachymeningitis

An unusual presentation of CNS TB is isolated involvement of the dura, known as pachymeningitis, which is distinct from the inflammation of the dura adjacent to an intraparenchymal tuberculoma.[20,21] It consists of either isolated dural involvement or pial or parenchymal involvement that is secondary to a dura-based lesion. As in the case of TBM, tuberculous pachymeningitis may also result from hematogenous spread of the bacilli. Pachymeningitis may exist as focal or diffuse involvement of the dura.[20,21] Focal pachymeningitis appears isointense on T1W, iso- to hypointense on T2W, and enhanced on postcontrast images. In contrast to focal lesions, diffuse involvement may appear hyperintense on T2W images. However, the MRI imaging findings of focal and diffuse pachymeningitis are nonspecific and may be seen in a large number of inflammatory and noninflammatory conditions.[20,21]

### Intracranial tuberculoma

Brain tuberculoma, a space-occupying mass of granulomatous tissue,[1] forms a large percentage of intracranial mass lesions in the developing countries and is responsible for high morbidity and mortality. Earlier recognition and treatment of this condition on imaging may play a critical role in patient management.

Tuberculomas may be single or multiple, and can be seen anywhere in the brain parenchyma. The number of identified lesions per patient may range from one to 12 (or more), with the size varying from 1 mm to 8 cm.[22] Its presence in the ventricular system is very rare. Although no precise patterns of localization have been observed according to race, age, or sex, children develop infratentorial tuberculomas more commonly than do adults.[23] Symptoms are often limited to seizures and mass effect, resulting in an increased intracranial pressure.[23] Neurological deficit reflects the topographic location of the lesion. These lesions originate as a conglomerate of microgranulomata in an area of tuberculous cerebritis that join to form a noncaseating tuberculoma. In most cases, subsequent central caseous necrosis develops that is initially solid, but in some instances, may eventually liquefy.[23]

**Figure 2(a-e):**
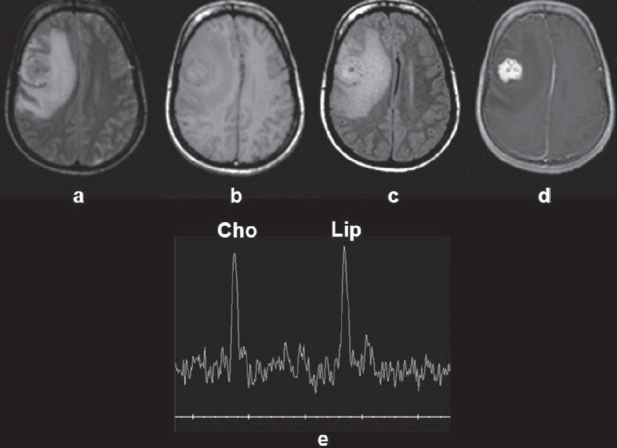
Atypical presentation of a right frontal tuberculoma in a 24-year-old female patient surgically excised due to nonresponse to therapy. The tuberculoma shows mixed signal intensity on the T2W image (a), slight hyperintensity on the T1W image (b), and hyperintensity on the magnetization transfer T1W image (c), with enhancement on the contrast-enhanced T1W image (d). Single-voxel magnetic resonance imaging spectroscopy (e) from the center of the lesion shows choline at 3.22 ppm and lipid at 1.3 ppm.

Intracranial tuberculomas usually show hypo- or isointensity or central hyperintensity with a hypointense rim on T2W images and isointensity and/or hypointensity on T1W images.[24–27] Certain tuberculomas show a varied range of signal intensities on MRI. Depending on its stage of maturation, a tuberculoma's appearance varies on MRI, *i.e*., whether noncaseating, caseating with a solid center, or caseating with a liquid center.[25,26] A noncaseating tuberculoma usually appears hyperintense on T2W and slightly hypointense on T1W images.[28] These granulomas show homogenous enhancement after injection of paramagnetic contrast on T1W images. A solid caseating tuberculoma appears relatively iso- to hypointense on both T1W and T2W images with an iso- to hyperintense rim on T2W images. In the presence of edema, the rim appears inseparable on T2W images. It shows rim enhancement on postcontrast T1W images. The degree of hypointensity of the solid caseating tuberculoma on T2W images depends on the complex relationship between the solid caseation, associated fibrosis/gliosis, macrophage infiltration, and perilesional cellular infiltrate. When the solid center of the caseating lesion liquefies, the center appears hyperintense with a hypointense rim on T2W images. The postcontrast T1W images show rim enhancement. MRI features of tuberculomas are known to overlap with those of other intracranial focal lesions, like the healing stage of neurocystycerecosis, fungal granulomas, chronic pyogenic brain abscess, and lymphomas. Some gliomas and metastases may also have features similar to those of tuberculomas and should be considered in their differential diagnoses.[23] Sometimes, large tuberculomas mimic neoplastic lesions on MRI as they appear predominantly hyperintense on T2W images, with mixed intensity on T1W images, and may show heterogeneous enhancement on postcontrast studies [Figure 2]. Quantitative MT imaging and *in vivo* proton MRS may help in the differential diagnosis of tuberculomas.[29–33]

**Figure 3(a-e):**
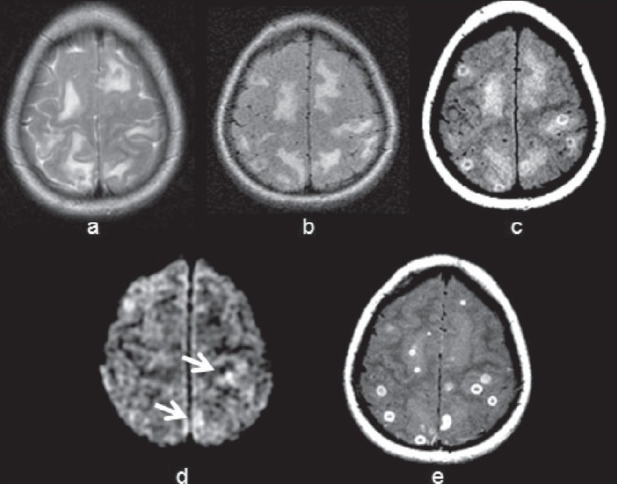
Multiple tuberculomas with perilesional edema exhibiting better definition of the wall on T1W magnetization transfer (MT) imaging in a 20-year-old man. T2W (a) and fluid-attenuated inversion recovery (b) images show areas of central hypointensity surrounded by edema. T1W MT image (c) shows conspicuous hyperintense walls in all lesions. DWI image (d) shows restriction in the core of some of the lesions (arrows). (e) Postcontrast T1W MT image shows ring as well as disc enhancement of all lesions.

Noncaseating tuberculomas show similar imaging features as in the case of metastases, lymphoma, and other infective granulomas.[28] On MT TIW imaging, cellular components of the lesions appear brighter and relatively specific for the disease. In addition, lesion conspicuity is greater on T1W MT imaging compared with conventional SE imaging and thus may help in improved assessment of the disease load. In solid caseating tuberculomas, hypointense solid caseation on T2W images often overlaps with the imaging features of lymphoma, glioblastoma, as well as fungal and cysticercus granulomas. On T1W MT images, the solid center appears hypointense, with a hyperintense rim. Calculated MTRs from the rim and the core are reported as 23.8 ± 1.76 and 24.2 ± 3.1, respectively.[34,35] The significantly lower MTR of the T2W hypointense tuberculoma compared with the cysticercus granuloma helps in its differential diagnosis. The lower MT ratio in different stages of tuberculoma is because of the high lipid content present in tuberculous bacteria. The fluid-attenuated inversion recovery (FLAIR) sequence has been reported to be useful in picking up more lesions, including brain infections. T1W MT imaging along with FLAIR imaging has been used to evaluate the conspicuity and the number of lesions in individuals with brain tuberculomas. FLAIR imaging has not been found to be useful in the examination of brain tuberculomas as compared with T1W MT imaging, as it neither contributes to the characterization of lesion nor assesses the true disease load.[36] Diffusion weighted imaging (DWI) shows restriction in tuberculomas with liquid necrosis [Figure 3], whereas there is no such restriction of diffusion in lesions with solid caseation. Restriction of diffusion in T2 hypointense lymphoma may differentiate it from tuberculoma.

**Figure 4(a-d):**
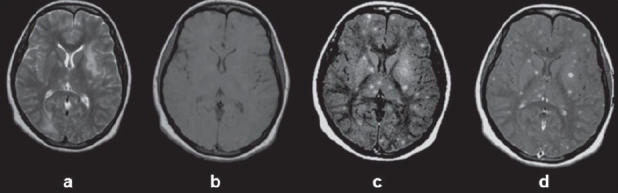
Miliary brain tuberculomas: T2W image (a) shows areas of hyperintensities in both cerebral hemispheres. Note a small hypointense lesion in the left globus palidus surrounded by perifocal edema. The corresponding T1W (b) axial image shows no definite lesion. Precontrast magnetization transfer T1W image (c) shows multiple hyperintense lesions in both cerebral hemispheres (more than on the T2W image), which show enhancement following contrast administration

Miliary brain tuberculosis is usually associated with TBM. Miliary tubercles are <2 mm in size and are either not visible on conventional SE MRI images or are seen as tiny foci of hyperintensity on T2W acquisitions. After gadolinium administration, T1W images show numerous, round, small, homogeneous, enhancing lesions. SE-invisible lesions that may or may not enhance after intravenous injection of gadolinium are clearly visible on MT-SE T1W imaging. MT-SE imaging helps in defining the true disease load [Figure 4].[10]

*In vivo* MRS is a powerful technique that can provide biochemical information of the pathophysiological process of the tissue under investigation. When combined with imaging, *in vivo* spectra are found to be specific for intracranial tuberculomas and demonstrate the biochemical fingerprints of tubercle bacilli in a granuloma. Gupta *et al*. have performed *in vivo*, *ex vivo*, and *in vitro* MRS to fingerprint the metabolites of *M. tuberculosis* in tuberculomas.[37] *In vivo* MRS with a stimulated echo acquisition mode (STEAM) sequence shows lipid resonances at 0.9, 1.3, 2.0, 2.8, and 3.7 ppm, corresponding to a terminal methyl group [ß(CH_3_)], methylene group ß(CH_2_)_n_, of a CH_2_¨CH fatty acyl chain, ¨CHßCH_2_CH¨ of a fatty acyl chain, and phosphoserine, respectively. SE sequences show a mark reduction in the peak intensities at 0.9 and 1.3 ppm peaks whereas the rest of the lipid signals are poorly visible. *Ex vivo* MRS of the excised tuberculomas confirms the resonances seen *in vivo*. Lipid peaks at 1.58, 2.24, 3.22, 4.1, 4.29, and 5.3 ppm are also seen on *ex vivo* MRS in addition to the signals seen *in vivo*, corresponding to OC ßCH_2_ ßCH_2_, and COCH_2_ of the fatty acyl chain, ßN(CH_3_)_3_ of Cho, and the glycerol backbone of phospholipids and olefinic groups of lipids, respectively. Signals of cyclopropane rings (0.5 and 0.1 ppm) and phenolic glycolipids (7.1–7.4 ppm) have been reported from the lipid extracts of a pure strain of *M. tuberculosis*. Phenolic lipids represent the biochemical fingerprint of *M. tuberculosis* in a granuloma; however, phenolic glycolipids remain present in the virulent as well as nonvirulent strains of *M. tuberculosis*. On *ex vivo* and *in vitro* (lipid extract) spectroscopy, peaks of cyclopropane rings and phenolic glycolipid caseating tuberculomas are seen, which can be attributed to *M. tuberculosis*.

*In vivo* spectroscopy shows only lipid in T2 hypointense tuberculomas, whereas lesions with a heterogeneous appearance show Cho at 3.22 ppm along with lipid. These lesions show a large amount of cellularity and minimal solid caseation, the cellular regions appearing brighter on MT imaging and showing Cho resonance on spectroscopy [Figure 2].

Dynamic contrast enhanced (DCE) MRI has been used for the purpose of *in vivo* quantification of angiogenesis in neoplastic lesions. In general, cerebral blood volume (CBV) provides information about the angiogenic activity of pathological tissue whereas permeability (k^trans^) and leakage (v_e_) give information related to the blood brain barrier integrity and changes in the extravascular–extracellular space.[38,39] Recently, Gupta *et al*. have performed DCE-MRI in 13 patients with brain tuberculomas and correlated the relative (r) CBV values with their cellular and necrotic components and also with the expression of immunohistochemical markers [microvascular density (MVD) and vascular endothelial growth factor (VEGF)].[40] rCBV of the cellular portion significantly correlates with cellular fraction volume, MVD, and VEGF of excised tuberculomas. MVD also correlates significantly with VEGF. Correlation among rCBV, MVD, and VEGF confirms that rCBV is a measure of angiogenesis in the cellular fraction of brain tuberculomas.[41] In a recent DCE-MRI study in brain tuberculomas, authors have reported a significant positive correlation between the physiological indices (k^trans^ and v_e_) and matrix metalloproteinase 9 (MMP-9) expression (a marker of BBB disruption) in excised tuberculomas. However, a weak correlation has been reported between physiological indices and VEGF expression in excised tuberculomas, suggesting a limited role of VEGF in leakage of the BBB. Correlation between k^trans^ and MMP-9 suggests that k^trans^ can be used as a surrogate marker of BBB disruption. Diffusion tensor MRI imaging (DTI) has been widely used for the detection of white matter abnormalities in various clinical conditions.[42–44] The degree of anisotropy, as measured by various anisotropy indices, is linked in some way to the integrity and density of orientated structures in the tissues.[45] The two most common DTI indices used for assessing white matter integrity are fractional anisotropy (FA) and mean diffusivity (MD). Other DTI indices, *i.e*., linear anisotropy (CL), planar anisotropy (CP), and spherical anisotropy (CS) modeled by Westin *et al.,* provide additional information associated with white matter (WM) integrity.[46] A recent serial DTI study has shown a strong negative correlation of MMP-9 expression in excised tuberculomas with FA, CL, and CP, and a significant direct correlation with CS. They have also reported a significant increase in FA, CL, and CP along with significantly decreased CS over time in patients who were serially followed-up with antituberculous therapy (ATT).[47]

### Tuberculous brain abscess

Tuberculous brain abscess is a relatively rare condition caused by *M. tuberculosis* infection, constituting 4–7% of the total number of CNS TB cases in developing countries. According to the criteria of Whitener, tuberculous abscesses should show macroscopic evidence of abscess formation within the brain parenchyma and should offer histological confirmation that the abscess wall is composed of vascular granulation tissue containing both acute and chronic inflammatory cells and *M. tuberculosis*.[48]

**Figure 5(a-g):**
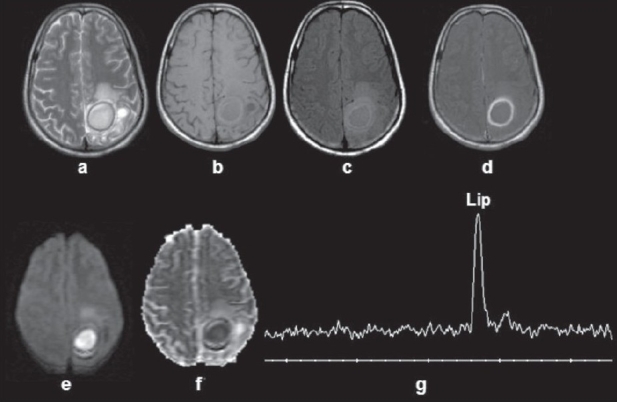
Tuberculous abscess in the left parasagittal region of a 20-year-old woman. A well-defined hyperintense lesion with a hypointense wall is seen in a T2W image (a). A T1W image shows a hypo- to isointense lesion with a hyperintense wall. A magnetization transfer (MT) T1W image (c) shows more conspicuity of the T2 hypointense wall as compared with the T1W image. A postcontrast MT T1W image (d) shows rim enhancement A diffusion-weighted image (e) shows homogeneous hyperintensity in the cavity with low ADC on the ADC map (f). Magnetic resonance imaging spectroscopy (G) from the center of the lesion with a voxel size of 1.2 ml shows a predominant lipid peak (Lip, 1.3 ppm)

Neuroimaging study findings are usually nonspecific for the detection of tuberculous abscesses. They appear as large, solitary, and frequently multiloculated, ring-enhancing lesions with surrounding edema and mass effect on MRI.[49] MTR quantification from the rim of the abscess has helped in the differential diagnosis of tuberculous from pyogenic abscesses.[50] High lipid-containing *M. tuberculosis* bacilli are probably responsible for the significantly lower MTR values in the rim of tuberculous abscesses (19.89 ± 1.55) compared with pyogenic abscesses (24.81 ± 0.03). DWI in tuberculous abscesses has shown restricted diffusion with low apparent diffusion coefficient (ADC) values, probably a result of the presence of intact inflammatory cells in the pus [Figure 5].[51–54]

*In vivo* MRS has also been used for the differentiation of tuberculous abscesses from other lesions such as pyogenic abscesses and fungal lesions. *In vivo* proton spectra in tuberculous abscesses show only Lac and lipid signals (at 0.9 and 1.3 ppm) without any evidence of cytosolic amino acids. More lipid peaks may also be apparent on *ex vivo* spectroscopy.

## Spinal TB

### Intraspinal TB

Spinal meningitis and spinal arachnoiditis are inflammatory spinal diseases caused by *M. tuberculosis*.[55] The pathophysiology of spinal meningitis is similar to that of TBM: a submeningeal tubercle forms during primary infection and ruptures into the subarachnoid space, eliciting mediators of delayed hypersensitivity.[55] As with intracranial lesions, there is granulomatous inflammation with areas of caseation and tubercles with eventual development of fibrous tissue in chronic or treated cases.

**Figure 6(a-c):**
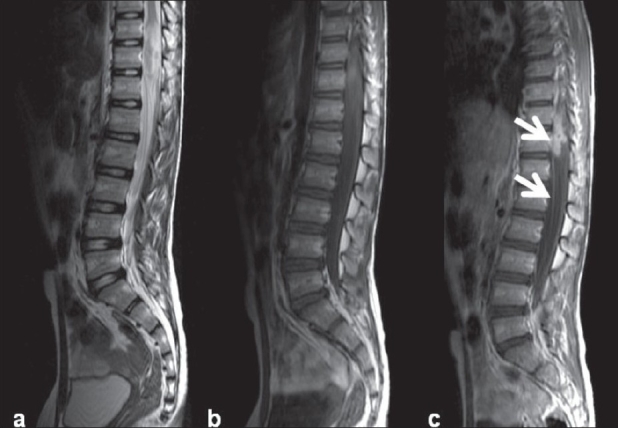
Intramedullary tuberculoma with arachnoiditis. Heterogeneous hyperintensity is seen at the conus on this T2W image (a), appearing hypo–isointense on a T1W image (b) and showing intense enhancement (arrow) on a postcontrast T1W image (c). Note the nerve roots as well as dural enhancement consistent with arachnoiditis (arrows)

MRI features include CSF loculation and obliteration of the spinal subarachnoid space with a loss of outline of the spinal cord in the cervicothoracic spine and matting of the nerve roots in the lumbar region. Sometimes, patients who appear normal on unenhanced MRI images may show nodular, thick, linear, intradural enhancement, often completely filling the subarachnoid space on postcontrast images.[56,57] In chronic stages of the disease, the postcontrast images may not show any enhancement even when unenhanced images show signs of arachnoiditis.[56,57] Spinal cord involvement in the form of infarction and syringomyelia may occur as a complication of arachnoiditis [Figure 6]. Parenchymal TB myelitis and tuberculoma formation may also occur.[56,57] Syringomyelia is seen as cord cavitation that typically demonstrates CSF intensity on T1W and T2W images but does not enhance on postcontrast images.[56,57]

#### TB myelitis:

The MRI imaging features of TB myelitis are similar to those of cerebritis. After 1 week of initiation of treatment, the region of myelitis becomes less diffusely hyperintense on T2W images, with more clearly defined marginal enhancement on postcontrast T1W images.[57,58] The surrounding edema continues to be more extensive than the margins of enhancement. These findings suggest the beginning of intramedullary abscess formation. The central cavitary portions of the intra-axial necrotic areas are seen as hypointense and hyperintense foci on T1W and T2W images, respectively.[58] Although the abnormalities visible on T2W images subside in several weeks, foci of contrast enhancement on postcontrast images may persist for several months.[58] *Dural and subdural pathology:* Tuberculous pus formation occurs between the dura and the leptomeninges and may appear loculated. It appears hyperintense on T2W and iso- to hypointense on T1W images. The dural granulomas appear hypo- to isointense on T2W and isointense on T1W images. Rim enhancement can be seen on postcontrast images.[57]

Epidural TB lesions generally appear to be isointense to the spinal cord on T1W images and have mixed intensity on T2W images. In postcontrast images, uniform enhancement can be seen if the TB inflammatory process is phlegmonous in nature whereas peripheral enhancement is seen if true epidural abscess formation or caseation has developed.[57,58] Epidural tuberculous abscess may occur as primary lesions or may be seen in association with arachnoiditis, myelitis, spondylitis, and intramedullary and dural tuberculomas.[57,58]

### Tuberculous spondylitis

Tuberculous spondylitis is an important cause of spinal disease in developing countries. Early diagnosis and prompt treatment are essential to avoid permanent damage or deformity in the spine.

**Figure 7(a-e):**
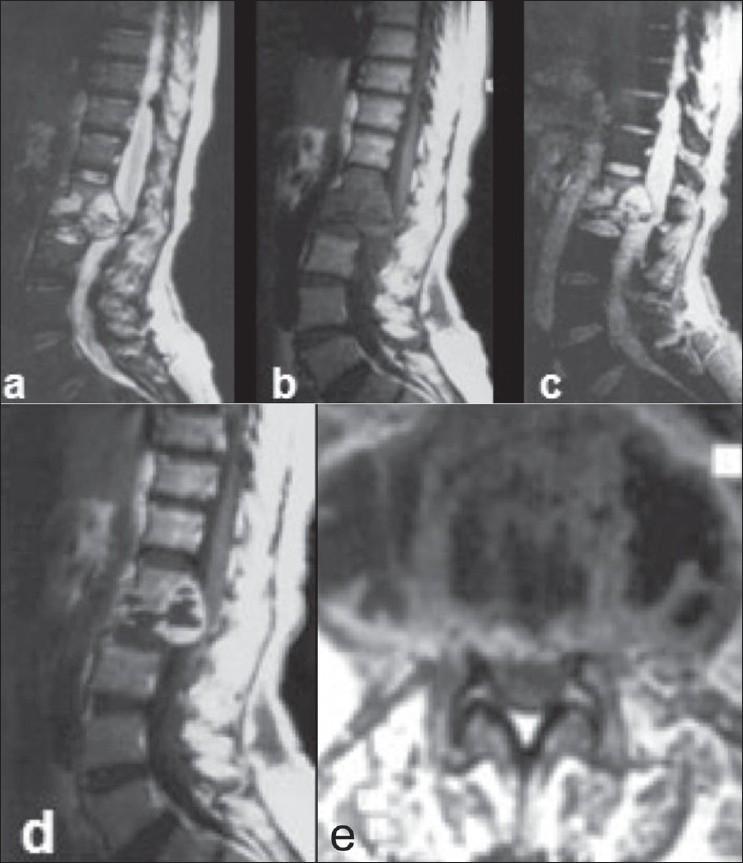
Tuberculous spondylitis: T2W sagittal image (a) shows a hyperintense destructive process involving the first and second lumbar vertebrae and the intervening disc. There is evidence of a hyperintense collection with areas of hypointensity, extending into the spinal canal and causing extradural compression. The vertebrae and collection appear hypointense on the T1Wimage (b) with a slightly hyperintense rim. Some of the hypointense areas visible on the T2W image show a susceptibility effect on the gradient echo image (c) consistent with bone fragments. Postcontrast T1W sagittal (d) and axial (e) images show peripheral enhancement of the lesion along with rim enhancement of the pre- and paravertebral collections

Tuberculous spondylitis involves one or more extradural components of the spine. The vertebral bodies are the most commonly involved; the posterior osseous elements, epidural space, paraspinal soft tissue, and intervertebral discs are also involved either secondarily or sometimes as the primary area to be first involved [Figure 7].[59] The most commonly involved sites are the dorsal and lumbar spine, especially the thoracolumbar junction. Although the sacrum and cervical spine are the least affected, more than one vertebral level is often involved.

Because of its ability to detect marrow abnormalities before bony destruction, MRI is sensitive for the early detection of tuberculous spondylitis, even in patients with normal radiographs. In the majority of cases, tuberculous spondylitis appears hyperintense on T2W and hypointense on T1W images, showing vertebral body involvement.[59,60] Classic disco-vertebral involvement can be noted with progression of the disease. Vertebral intra-osseous abscesses, paraspinal abscesses, discitis, skip lesions, and spinal canal encroachment are all readily seen on routine SE imaging. Reduction in disc height and morphological alteration of the paraspinal soft tissue become apparent during the later stages of infection.

**Figure 8(a-g):**
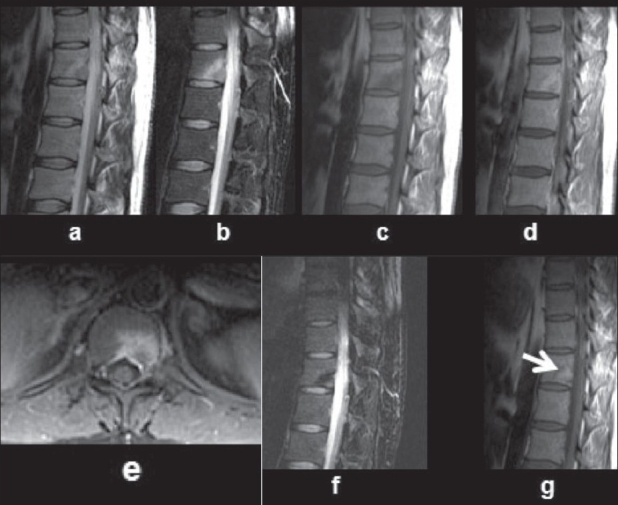
Early tuberculous spondylitis in a young adult male: T2W sagittal image of the dorso-lumbar spine with (a) and without (b) fat suppression shows a hyperintense lesion in the posteromedial part of the 12th dorsal vertebra, better appreciated in 8b. The lesion appears hypointense on the T1W image (c) and shows enhancement on the postcontrast T1W image (d). The axial postcontrast, T1W, fatsuppressed image clearly defines the enhancing lesion (e). A repeat study after 4 months of antituberculous therapy shows resolution of the hyperintensity (f) and replacement of the hypointense lesion by fat (arrow) on the T1W image (g), consistent with healing.

Hyperintensity in involved vertebral bodies, discs, and soft tissues on T2W images reflect inflamed and edematous structures. The internuclear cleft within the disc is a normal finding in individuals above 30 years of age. Its loss on imaging in combination with a prolonged T2 value can be a sign of inflammation.[60] Enhanced MRI studies are useful for characterizing tuberculous spondylitis. Rim enhancement on postcontrast T1W images around intra-osseous and paraspinal soft tissue abscesses is characteristic of tuberculous spondylitis.[60] Although rim enhancement classically suggests abscess formation, it may also be seen in solid caseating tuberculomas.[60] During chemotherapy, a progressive increase in signal intensity on T1W images in previously affected vertebrae suggests fatty marrow replacement and healing [Figure 8].[59]

CT scan demonstration of bone fragments in the intra- and/or extraspinal soft tissue has been described as being characteristic of tuberculous spondylitis.[59] This feature is attributable to the lack of proteolytic enzymes required to lyse bone in the tuberculous inflammatory exudate. CT scan is considered superior to MRI for the demonstration of these small bone fragments. T2*W images have been shown to better demonstrate calcification compared with SE images by accentuating the diamagnetic susceptibility properties of calcium salts. The low signal is more prominent on T2*W than on SE images and closely matches the calcification seen on CT. Demonstration of bone fragments on T2*W images is also considered to be characteristic of tuberculous spondylitis even in the absence of abscess formation.[60]

### Therapeutic response assessment

**Figure 9(a-j):**
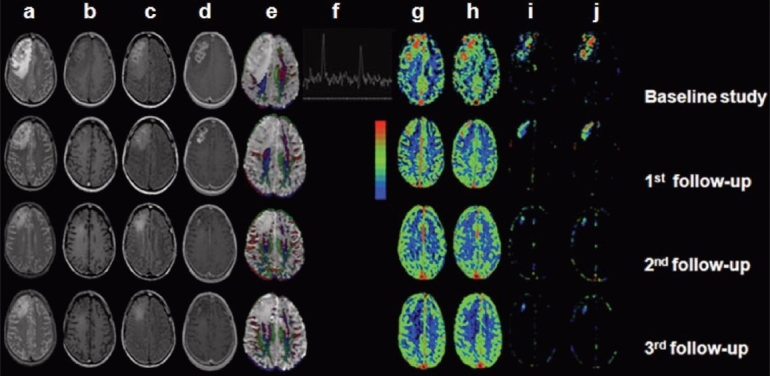
Right frontal tuberculoma in a 50-year-old male patient on antituberculous therapy with follow-up every 4 months for a total period of 1 year. The tuberculoma shows mixed intensity on the T2W images (a), slight hyperintensity on the T1W images (b), and hyperintensity on the magnetization transfer (MT) T1W images (c), with enhancement on the immediate contrast-enhanced T1W images (d) in all the studies. The hyperintense portion of the lesion is cellular with a very small necrotic fraction appearing hypointense on the MT T1W images. The cellular and total volumes of the lesion are seen to gradually decrease over 1 year on serial imaging. This is associated with a reduction in edema (T2W images) at each study time point. Color-coded fractional anisotropy (FA) map (e) shows gradual recovery of FA values with time in white matter regions. The perfusion indices [i.e., cerebral blood volume (f), cerebral blood flow (CBF) (g), ktrans (h), and ve (i) map] from the corresponding sections reveal a gradual decrease in their respective values at each time point over 1 year.

MRI is the modality of choice for following the benefits of treatment in patients with CNS tuberculosis. Most patients are treated with ATT after the diagnosis is suggested by imaging and other laboratory investigations.[25,61] However, the duration of medical treatment is largely empirical and is based on data from a small number of publications.[25,61] Serial imaging in patients on ATT may show a decrease in the lesion size within 3 and 4 months and complete disappearance at the end of 12 months.[61] Paradoxical progression of intracranial tuberculomas or development of new lesions during treatment has been recognized as a rare response to ATT. Using DCE-MRI, it has been shown that changes in k^trans^ and v_e_ are associated with therapeutic response even in the presence of a paradoxical increase in the lesion volume [Figure 9].[41]

**Figure 10(a-j):**
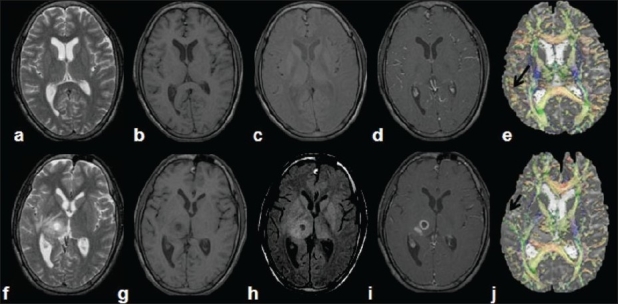
Tuberculous meningitis with tuberculoma. T2W (a) as well as T1W (b) images show no apparent changes in the brain parenchyma. Magnetization transfer (MT) T1W (c) and postcontrast T1W (d) images in the initial study show meningeal enhancement (arrows). Color-coded fractional anisotropy (FA) map modulated by the principal eigenvector (e) shows increased FA values (arrow) in the cerebral cortical region that showed meningeal enhancement. After 3 months of antituberculous treatment, the patient developed a lesion in the right thalamic region, visible on T2W (f), T1W (g), MT T1W (h), and postcontrast T1W (i) images, consistent with a tuberculoma. The abnormalities in the subcortical region (arrow) appear to have reversed on the postcontrast T1W image (i) as well as on the color-coded FA maps (j)

Less-intense meningeal enhancement on postgadolinium MRI studies following ATT in patients with TBM is considered as a response to treatment. A recent serial DTI study in TBM has reported increased FA values in cerebral cortical regions in TBM patients (0.15 ± 0.03) as compared with controls (0.10 ± 0.02) at the time of the baseline study [Figure 10]. On follow-up after 3 months of ATT, these regions in the TBM patients have shown significantly decreased FA values (0.13 ± 0.02) compared with the initial study in the entire cerebral cortical region as well as basal meninges. They have reported a significant positive correlation between FA and proinflammatory molecules (PMs), and suggested that DTI metrics may be used as a noninvasive surrogate marker of PMs in TBM in assessing therapeutic response.

We conclude that conventional imaging supplemented by advanced MRI techniques helps in improved tissue characterization of CNS tuberculosis and may help in better management of these patients.
